# Expression patterns of MRP2 in circulating tumor cells of breast cancer: a single-institution study

**DOI:** 10.3389/fonc.2025.1648842

**Published:** 2025-09-23

**Authors:** Jiayu Guan, Fuping Li, Wenbin Zhou

**Affiliations:** ^1^ Department of Breast Surgery, Shenzhen People's Hospital (The First Affiliated Hospital, Southern University of Science and Technology; The Second Clinical Medical College, Jinan University), Shenzhen, China; ^2^ Department of Surgery, Second Affiliated Hospital of School of Medicine, Zhejiang University, Hangzhou, Zhejiang, China

**Keywords:** MRP2, breast cancer, circulating tumor cells, EMT, multiplex RNA *in situ* analysis

## Abstract

**Background:**

Breast cancer metastasis remains a major oncology challenge, with circulating tumor cells (CTCs) driving dissemination and multidrug resistance (MDR) hindering treatment efficacy. MRP2, an ABC transporter linked to MDR, may promote CTC survival; however, its expression in CTCs and its association with epithelial-mesenchymal transition (EMT) in breast cancer remain underexplored.

**Materials and methods:**

A total of 52 breast cancer patients were recruited for the study, from whom circulating tumor cells (CTCs) were isolated from 5 ml of peripheral blood samples utilizing the CanpatrolTM CTC detection platform. Subsequently, a comprehensive multiple mRNA *in situ* analysis (MRIA) employing diverse molecular markers was conducted to accurately identify and categorize CTCs. The relationships between CTC counts, subtypes (epithelial type, E type; hybrid epithelial/mesenchymal type, H type; mesenchymal type, M type), and MRP2 expression in CTCs were analyzed using Spearman’s correlation coefficient. Statistical analyses were performed using the SPSS software.

**Results:**

CTCs were detected in 94.2% of patients. H-type CTCs and MRP2 (+) CTCs were significantly associated with larger tumor size (*P* < 0.05). MRP2 expression was higher in (H+M)-type than in E-type CTCs (*P* < 0.001). EMT grade was positively correlated with MRP2 (+) CTCs grade and high MRP2 expression (*R* = 0.283, *P* = 0.042), with strong correlations between all CTC subtypes and MRP2 expression.

**Conclusion:**

This study pioneers the MRP2-CTCs-EMT axis in breast cancer, clarifying MRP2’s role in CTC biology and EMT, providing a theoretical basis for combined targeting strategies to improve metastatic breast cancer management.

## 1 Introduction

Breast cancer metastasis remains a significant challenge in oncology, primarily due to its complex biology and the mechanisms that enable tumor cells to disseminate and establish secondary tumors (1, 2). Among the various factors contributing to metastasis, circulating tumor cells (CTCs) play a pivotal role, as they detach from the primary tumor, enter the bloodstream, and eventually colonize distant organs (3). The presence of CTCs is associated with poor prognosis, as these cells are often more resistant to conventional therapies, complicating treatment strategies (4). The mechanisms underlying CTC survival and dissemination are multifaceted and involve various cellular processes and molecular pathways that remain to be fully elucidated.

One critical aspect of breast cancer metastasis is the role of drug efflux transporters, particularly the multidrug resistance-associated protein 2 (MRP2, also known as ABCC2) (5). MRP2 is a member of the ATP-binding cassette (ABC) transporter family, which actively transports a wide range of substrates, including chemotherapeutic agents, out of cells (6). This efflux mechanism is a significant contributor to the phenomenon of multidrug resistance (MDR), where cancer cells develop the ability to evade the cytotoxic effects of chemotherapy (7). The overexpression of MRP2 in CTCs has been linked to enhanced cell survival and increased metastatic potential, suggesting that targeting this transporter could be a promising strategy for improving treatment outcomes in patients with breast cancer (8).

Recent studies have highlighted the correlation between MRP2 expression in CTCs and their ability to survive in the circulatory system. High levels of MRP2 have been observed in CTCs from patients with breast cancer, indicating that these cells may utilize MRP2-mediated efflux as a mechanism to evade drug-induced apoptosis (9). Furthermore, the interaction between MRP2 and other signaling pathways, such as those involving EMT, further complicates the landscape of breast cancer metastasis (10). EMT is a process that allows epithelial cells to acquire migratory and invasive properties, and it has been shown to enhance the expression of MRP2, thereby facilitating the survival of CTCs and their metastatic spread (11).

The clinical significance of MRP2 in breast cancer is underscored by its potential as a therapeutic target for breast cancer. By understanding the regulatory mechanisms governing MRP2 expression and activity, new strategies can be developed to inhibit its function, thereby increasing the sensitivity of CTCs to chemotherapeutic agents. Moreover, research on the modulation of MRP2 activity by dietary components or pharmacological agents may provide additional avenues for enhancing treatment efficacy (12). Nevertheless, to date, there has been no investigation into the expression levels of MRP2 in CTCs. Furthermore, the association between MRP2 expression and the EMT process in CTCs derived from breast cancer has yet to be elucidated. Therefore, a comprehensive understanding of MRP2’s role in CTC biology is essential for developing novel therapeutic approaches aimed at reducing the metastatic burden in patients with breast cancer.

Given these findings, exploring the MRP2-CTCs relationship is highly significant, as both factors are related to breast cancer metastasis and prognosis. Investigating MRP2 expression in CTCs and its intrinsic link could elucidate the mechanisms underlying breast cancer metastasis and drug resistance. This finding could provide a theoretical basis for combined CTCs-MRP2 targeting strategies. The insights gained from this study could pave the way for innovative treatments that specifically target the pathways involved in CTC-mediated metastasis, ultimately improving patient outcomes in breast cancer management.

## 2 Materials and methods

### 2.1 Patients and blood samples collection

A total of 57 confirmed breast cancer cases treated at Shenzhen People’s Hospital (China) from September 2022 to May 2024 were selected for CTCs detection and MRP2 protein quantification. Ethical approval for the peripheral blood study was obtained from the Ethics Committee of the Shenzhen People’s Hospital (Shenzhen, China; ethics approval number: LL-KY-2024078-01). The clinical characters of 52 patients (five patients were excluded because of incomplete clinical information) were collected, including age, gender, tumor size, ER, PR, HER-2, and other clinicopathological features. The exclusion criteria were defined as follows: 1) incomplete clinical information (e.g., missing pathological subtype, tumor stage, or biomarker status [ER/PR/HER2]); 2) concurrent diagnosis of other malignant tumors (to avoid CTC interference from non-breast cancer sources); 3) severe hepatic or renal dysfunction (estimated glomerular filtration rate <30 mL/min/1.73m² or Child-Pugh Class C), as organ dysfunction may affect CTC survival in circulation; 4) history of hematological diseases (e.g., leukemia, lymphoma) that could confound leukocyte depletion during CTC isolation; 5) inability to cooperate with peripheral blood collection (e.g., severe coagulation disorders). The data on breast cancer type and stratification by therapy received of these patients are shown in **Supplementary Table 1**.

In order to prevent contamination of cells resulting from puncturing the skin veins, the initial 2 ml of peripheral blood was discarded. Subsequently, 5 ml of blood was collected into an ethylenediaminetetraacetic acid (EDTA) tube (Becton Dickinson, Shanghai, China). The Canpatrol System (SurExam Biotech, Guangzhou, China) was used for analysis within 4h after blood collection.

### 2.2 Isolation of CTCs from peripheral blood

The previously reported Canpatrol platform was used to enrich and identify CTCs from blood (13). First, collect 5 ml of peripheral blood sample from the patient using an EDTA tube, invert, and mix well. Next, 15 ml of red blood cell lysis buffer (154 mM NH4Cl, 10 mM KHCO3, and 0.1 mM EDTA) was added, mixed again, and allowed to stand at room temperature for 30 min to lyse red blood cells. Subsequently, the sample at 500 g for 5 min, the supernatant was removed, and the cell pellet was resuspended in PBS. Next, the remaining cell pellet was fixed with 4% formaldehyde (final concentration) for 8 min. After fixation, the cells were transferred to a filter tube containing a filter membrane (SurExam Biotech, Guangzhou, China) with a pore size of 8 µm, and the cells were filtered onto the membrane using a vacuum filtration pump (Auto Science, Tianjin, China). Finally, the filtered cell membrane sample was fixed with 4% formaldehyde at room temperature for 1 h.

### 2.3 Multiplex RNA *in situ* analysis detection methods

The fixed membrane samples were washed three times with PBS and placed in a 24-well plate. Proteinase K (0.1 mg/mL; Qiagen, Hilden, Germany) was added for treatment, and the samples were left to stand at room temperature for 1 h to increase cell membrane permeability. The samples were then washed three times with PBS, followed by the addition of specific capture probes for hybridization, including epithelial biomarkers (EpCAM, CK8/18/19), mesenchymal biomarkers (vimentin and twist), and the leukocyte marker CD45. The probes were synthesized by Shanghai Sangon Bioengineering Company (Shanghai, China), and the probe sequences are listed in **Table 1**. The hybridization reaction was performed at 40 °C for 3 h. Unbound probes were washed three times with 1000 μl of eluent (formulation: 0.1×SSC (Sigma, St. Louis, USA)). Subsequently, a volume of 100 μl of the pre-amplification solution was introduced, comprising 30% horse serum (Sigma, St. Louis, USA), 1.5% sodium dodecyl sulfate (Sigma, St. Louis, USA), 3 mM Tris-HCl (pH 8.0), and 0.5 fmol of pre-amplification probes as detailed in **Table 2**. The samples were then incubated at 40 °C for 30 min to facilitate the reaction involving the signal amplification probes. This process involved the conjugation of capture probes to branched DNA (b-DNA) signal amplification probes, resulting in the formation of a branched structure. After cooling the membrane, it was washed three times with 1000 μl of 0.1×SSC eluent, then incubated with 100 μl of amplification solution (containing 30% horse serum, 1.5% sodium dodecyl sulfate, 3 mM Tris-HCl (pH 8.0), and 1 fmol pre-amplification probes (**Table 2**)) at 40 °C for 30 min.

**Table 1:** Capture probe sequences.

Gene	Sequence (5’ - 3’)	
EpCAM	TGGTGCTCGTTGATGAGTCA	AGCCAGCTTTGAGCAAATGA
	AAAGCCCATCATTGTTCTGG	CTCTCATCGCAGTCAGGATC
	TCCTTGTCTGTTCTTCTGAC	CTCAGAGCAGGTTATTTCAG
CK8	CGTACCTTGTCTATGAAGGA	ACTTGGTCTCCAGCATCTTG
	CCTAAGGTTGTTGATGTAGC	CTGAGGAAGTTGATCTCGTC
	CAGATGTGTCCGAGATCTGG	TGACCTCAGCAATGATGCTG
CK18	AGAAAGGACAGGACTCAGGC	GAGTGGTGAAGCTCATGCTG
	TCAGGTCCTCGATGATCTTG	CAATCTGCAGAACGATGCGG
	AAGTCATCAGCAGCAAGACG	CTGCAGTCGTGTGATATTGG
CK19	CTGTAGGAAGTCATGGCGAG	AAGTCATCTGCAGCCAGACG
	CTGTTCCGTCTCAAACTTGG	TTCTTCTTCAGGTAGGCCAG
	CTCAGCGTACTGATTTCCTC	GTGAACCAGGCTTCAGCATC
Vimentin	GAGCGAGAGTGGCAGAGGAC	CTTTGTCGTTGGTTAGCTGG
	CATATTGCTGACGTACGTCA	GAGCGCCCCTAAGTTTTTAA
	AAGATTGCAGGGTGTTTTCG	GGCCAATAGTGTCTTGGTAG
Twist	ACAATGACATCTAGGTCTCC	CTGGTAGAGGAAGTCGATGT
	CAACTGTTCAGACTTCTATC	CCTCTTGAGAATGCATGCAT
	TTTCAGTGGCTGATTGGCAC	TTACCATGGGTCCTCAATAA
CD45	TCGCAATTCTTATGCGACTC	TGTCATGGAGACAGTCATGT
	GTATTTCCAGCTTCAACTTC	CCATCAATATAGCTGGCATT
	TTGTGCAGCAATGTATTTCC	TACTTGAACCATCAGGCATC
MRP2	GATTAGAATTGTCACCCTGT	TGCACAGAGATATCCAATCC
	AATGGTCTTACTCTTGGTGG	TCTCATCCACTTGAGGAAGA
	CCAGAGGTTGGATCCAATAA	GCATGGACGAAACCAAAGGC
	CCACAATGTTGGTCTCTATT	ACTCTATAATCTTCCCGTTG

**Table 2:** Sequences for the bDNA signal amplification probes.

Probe tapes	Function (copies)	Sequence (5’-3’)	Complement
bDNA probes for EpCAM and CK8/18/19	Capture probe tail	CTACAAACAAACAATATT	Preamplifer leader
	Preamplifer repeat	CGCAGCCTCAGCC	Amplifer leader
	Amplifer repeat	CCCAGACCCTACC	Label probe
bDNA probes for vimentin and twist	Capture probe tail	CTTCTCAATAACTAACAT	Preamplifer leader
	Preamplifer repeat	GACGGTCGGCGTT	Amplifer leader
	Amplifer repeat	GTCACCGCTCCAC	Label probe
bDNA probes for CD45	Capture probe tail	GTAAAAAGAAAGGTATAA	Preamplifer leader
	Preamplifer repeat	AATTATACATCTC	Amplifer leader
	Amplifer repeat	GAAATGAATGAAT	Label probe
bDNA probes for MRP2	Capture probe tail	CTTTATACCTTTCTTTCA	Preamplifer leader
	Preamplifer repeat	GCGCGCTGTAGGG	Amplifer leader
	Amplifer repeat	AGGCGAGGGGAGA	Label probe

b-DNA, branched DNA.

**Figure 1:**
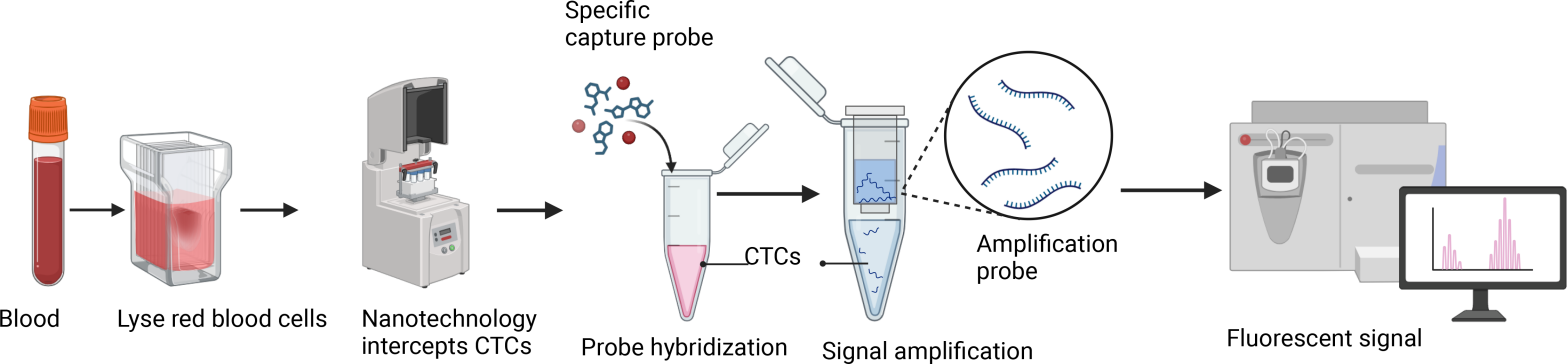
Flowchart of CTCs detection.

Subsequently, three fluorescent protein-labeled probes (Shanghai Sangon Bioengineering Company, Shanghai, China) (**Table 2**) were added: Alexa Fluor 594 (for labeling epithelial biomarkers EpCAM, CK8/18/19), Alexa Fluor 488 (for mesenchymal biomarkers vimentin and twist), Alexa Fluor 750 (for leukocyte marker CD45), and Alexa Fluor 647 (for MRP2 mRNA), followed by incubation at 40 °C for 30 min. Finally, the samples were eluted with 0.1×SSC and stained with 4’, 6-diamidino-2-phenylindole (DAPI) (Louis, USA) for nuclear staining for 5 min and observed under a 100× oil immersion lens using an automated fluorescence scanning microscope (ZEISS, Germany). Red and green fluorescent signal dots represent the expression of epithelial and mesenchymal genes in CTCs, respectively, while white signal dots represent the gene expression of the leukocyte marker CD45. The purple signal dots represent MRP2 expression (a cut-off point was set at two signal dots: “low expression” and “high expression”). The CTCs classification criteria are shown in **Table 3**. The study flowchart is shown in **Figure 1**.

**Table 3:** CTCs classification.

	Types	Red fluorescent	Green fluorescent	White fluorescent	DAPI
CTCs	Epithelial type	+	–	–	+
	Hybrid epithelial/mesenchymal type	+	+	–	+
	Mesenchymal type	–	+	–	+

CTCs, circulating tumor cells; DAPI, 4’, 6-diamidino-2-phenylindole.

### 2.4 Statistical analysis

Data were analyzed using SPSS software (version 21.0; SPSS Inc.). Continuous variables were documented as medians accompanied by their respective ranges, while categorical variables were expressed in terms of frequency and percentage. Patients were divided into two groups according to high and low CTC/cluster numbers and high and low gene expression levels. The chi-square test was employed to assess the associations between clinicopathological characteristics and the expression of CTCs or MRP2 expression in CTCs. Statistical significance was set at P < 0.05.

## 3 Results

### 3.1 Characteristics of patients and detection of CTCs

The clinical characteristics and CTCs detection data of the 52 patients with breast cancer are shown in **Table 4**. Most patients were female (98.1%), with a median age of 47 years (range: 34–72 years; 11.5% < 40 years). The median tumor size was 1.5 (range: 0.2-6), and 79% had tumors ≤ 2. The pathological stage had a median of 1 (range: 0-4), with 76.9% of patients at stage ≤ II.

**Table 4:** Clinical characteristics and CTCs detection of the 52 breast cancer patients.

Pathological paramenters		Median	Range	N	Percentage (%)
Total cases				52	
Gender		N/A	N/A		
	Male/Female			1/51	1.9/98.1
Age		47	34-72		
	< 40/≥ 40			6/46	11.5/88.5
Tumor size		1.5	0.2-6		
	≤ 2/> 2			39/13	79/25
Pathological stage		1	0-4		
	≤ II/> II			40/12	76.9/23.1
CTCs count		15	0-108		
	< 1/≥ 1/5ml			3/49	5.8/94.2
E type CTCs		3.5	0-63		
	< 1/≥ 1/5ml			12/40	23.1/76.9
H type CTCs		7.5	0-43		
	< 1/≥ 1/5ml			6/46	11.5/88.5
M type CTCs		0	0-5		
	< 1/≥ 1/5ml			46/6	88.5/11.5
MRP2 (+) CTCs		13	0-84		
	≤ 13/> 13/5ml			24/25	48.9/51.1

CTCs, circulating tumor cells; MRP2 (+), MRP2 signal dots ≥ 1. N/A, not available.

**Figure 2:**
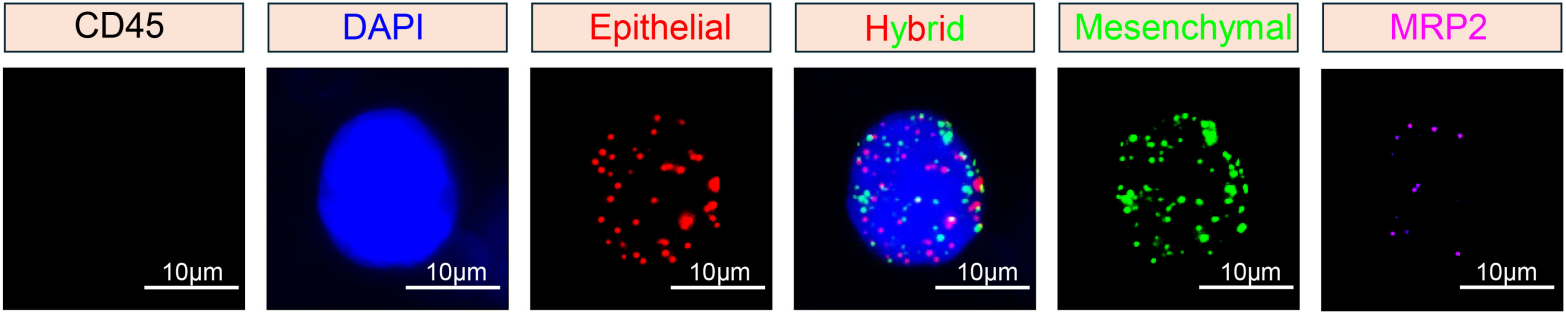
Representative images of CTCs with multiple mRNA *in situ* analysis.

CTCs were detected (≥ 1/5 ml) in 94.2% (49/52) of the breast cancer patients (**Table 4**). The median number of CTCs was 15 (range: 0-108) in 5 ml peripheral blood samples from all 52 patients. Using the Canpatrol™ CTC detection platform, all the separated CTCs were classified into three distinct EMT categories: E, H, and M types, utilizing various labeled mRNA probes. As illustrated in **Figure 2**, E-type cells correspond to the epithelial type, H-type cells denote the hybrid epithelial/mesenchymal type, and M-type cells are indicative of the mesenchymal type. E-type CTCs showed a median of 3.5 (range: 0-63), with 76.9% having ≥ 1/5 ml. H-type CTCs had a median of 7.5 (range: 0-43), and 88.5% had ≥ 1/5 ml. M-type CTCs had a median of 0 (range: 0-5), with 88.5% having < 1/5 ml. MRP2 (+) CTCs (MRP2 signal dots ≥ 1) had a median of 13 (range: 0-84), with 48.9% having ≤ 13/5 ml.

### 3.2 Correlation between CTCs and clinical features

The association between CTCs and clinical variables in 52 patients with breast cancer is presented in **Table 5**. For gender (1 male, 51 females), all CTC-related types (total, E, H, M, H + M, MRP2 (+)) showed no significant differences (*P* > 0.05, e.g., total CTCs: median 23 vs. 15 (0–108), *P* = 0.654). In terms of age (< 40 years: 6 patients; ≥ 40 years: 46 patients), no significant differences were observed for all CTC-related types (*P* > 0.05, e.g., total CTCs: median 26.5 (0-108) vs 14 (0-39), *P* = 0.332). For tumor size (≤ 2:39 patients; > 2:13 patients), significant differences were found in H-type CTCs (median 6 (0-30) vs 15 (2-43), *P* = 0.044) and MRP2 (+) CTCs (median 12 (0-35) vs 17 (3-84), *P* = 0.039), suggesting that CTCs may play an important role in the progression of breast cancer. Other CTC-related parameters (total, E, M, and H + M) showed no significant differences (*P* > 0.05). Regarding pathological stage (≤ II: 40 patients; > II: 12 patients), no significant differences were detected for all CTC-related types (*P* > 0.05, e.g., total CTCs: median 14 (0-108) vs 19 (6-37), *P* = 0.192). Overall, only H-type and MRP2 (+) CTCs exhibited significant differences between the different tumor sizes. Most CTC-related types showed no significant associations with gender, age, or pathological stage.

**Table 5:** Association between CTCs and clinical variables in 50 breast cancer patients.

	N	Total CTCs		E type CTCs		H type CTCs		M type CTCs		H+M CTCs		MRP2 (+) CTCs	
	52	Median	*P*	Median	*P*	Median	*P*	Median	*P*	Median	*P*	Median	*P*
Gender													
Male	1	23	0.654	5	0.808	16	0.50	2	0.115	18	0.50	13	1.000
Female	51	15 (0-108)		3 (0-63)		7 (0-43)		0 (0-5)		7 (0-45)		13 (0-84)	
Age													
< 40	6	26.5 (0-108)	0.332	6 (0-63)	0.547	20 (0-43)	0.291	0.5 (0-3)	0.089	20.5 (0-45)	0.265	19 (0-84)	0.605
≥40	46	14 (0-39)		3.5 (0-19)		6.5 (0-35)		0 (0-5)		6.5 (0-35)		13 (0-36)	
Tumor size													
≤2	39	13 (0-61)	0.074	4 (0-28)	0.865	6 (0-30)	0.044	0 (0-5)	0.675	6 (0-33)	0.054	12 (0-35)	0.039
>2	13	22 (3-108)		3 (0-63)		15 (2-43)		0 (0-2)		15 (2-45)		17 (3-84)	
Pathological stage													
≤II	40	14 (0-108)	0.192	3.5 (0-63)	0.677	6.5 (0-43)	0.188	0 (0-5)	0.159	6.5 (0-45)	0.199	13 (0-84)	0.207
>II	12	19 (6-37)		5 (0-16)		10.5 (2-26)		0 (0-0)		10.5 (2-26)		16.5 (3-35)	

### 3.3 The expression of MRP2 in CTCs

The mRNA expression of MRP2 was detected using the MRIA assay (**Table 6**). The results showed that the MRP2 gene was expressed in 94.2% (49/52) of the CTC-positive patients. The median number of MRP2-positive CTCs was 2 in 5 ml of blood samples from patients. Further study showed that the expression rates of MRP2 in different types of CTCs were different: 76.3% (271/355) in the E-type CTCs and 85.9% (505/588) in the (H+M)-type CTCs. The difference in MRP2 expression between the two groups was statistically significant (*P* < 0.001; **Table 6**).

**Table 6:** The expression rates of the MRP2 gene in different types of CTCs.

CTCs type	NO. of CTCs	MRP2 (+) CTCs		MRP2 (-) CTCs		χ² test	
		N	Percentage (%)	N	Percentage (%)	χ²	P
E	355	271	76.3	84	23.7	13.8	<0.001
H+M	588	505	85.9	83	14.1		

### 3.4 MRP2 expression correlates with tumor size

An analysis was conducted to investigate the correlation between the expression levels of MRP2 in circulating tumor cells (CTCs) and the clinical characteristics of the patients, as presented in **Table 7**. The median count of MRP2-positive CTCs was determined to be 13 per 5 ml of blood samples, leading to the stratification of patients into two distinct groups: those with >13 MRP2-positive CTCs and those with ≤13 MRP2-positive CTCs per 5 ml of blood. A significant association was observed between the presence of >13 MRP2-positive CTCs and tumor size, in contrast to those with ≤13 MRP2-positive CTCs (*P* < 0.001). However, no significant differences were noted concerning other clinicopathological characteristics (*P* > 0.05).

**Table 7:** Relationship between MRP2 expression in CTCs and clinical pathological features.

Pathological parameters		N	MRP2 expression		χ² test	
			≤13 MRP2 positive CTCs	>13 MRP2 positive CTCs	χ²	P
Total cases		52	27	25		
Gender	Male	1	1	0	1.569	1.000
	Female	51	26	25		
Age	<40	6	2	4	4.322	0.411
	≥40	46	25	21		
Tumor size	≤2	39	23	16	3.107	0.048
	>2	13	4	9		
Pathological stage	≤2	40	22	18	0.657	0.417
	>2	12	5	7		
ER	+	36	21	15	1.926	0.165
	–	16	6	10		
PR	+	30	17	13	0.639	0.424
	–	22	10	12		
Her-2	+	10	6	4	2.234	0.729
	–	42	21	21		
Ki-67	≥20%	21	11	10	0.003	0.957
	<20%	31	16	15		

ER, estrogen receptor; PR, progesterone receptor; Her-2, human epidermal growth factor-2.

### 3.5 Correlation between EMT grade and MRP2 expression grade

**Figure 3:**
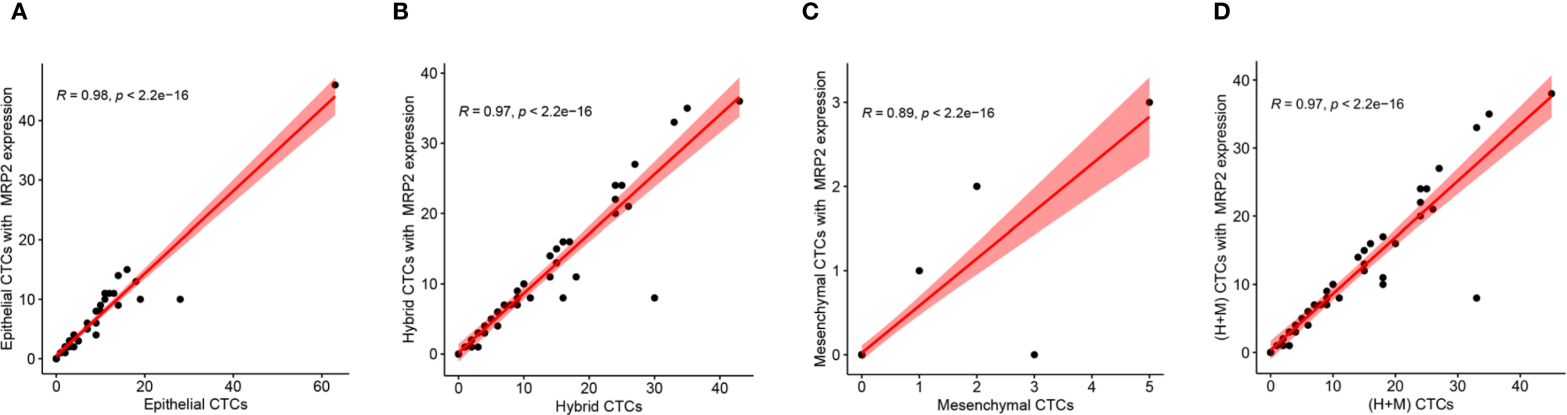
Correlation between MRP2 expression and different CTCs subtypes. Scatter plots show the positive relationships between MRP2 expression and the counts of **(A)** Epithelial CTCs, **(B)** Hybrid CTCs, **(C)** Mesenchymal CTCs, and **(D)** (H+M) CTCs. Red lines represent linear regression fits, with shaded areas indicating 95% confidence intervals. Pearson correlation coefficients (R) and *P*-values are displayed for each analysis, demonstrating significant positive correlations.

EMT levels were classified based on the ratio of (mixed-type + mesenchymal cell phenotypes) to total cells, divided into four grades: grade 1 (0-24%), grade 2 (25%-49%), grade 3 (50%-74%), and grade 4 (75-100%). According to the expression level of the MRP2 protein, the patients were divided into four groups. When the number of MRP2 expressions in CTCs is zero, it is negative; when it is greater than zero, it is positive. In CTCs, if the number of MRP2 signal points is ≤ 2, it is low expression; if it is > 2, it is high expression. The expression level of MRP2 was divided into four grades: 0-24% was grade 1, 25%-49% was grade 2, 50%-74% was grade 3, and 75-100% was grade 4. Spearman’s rank correlation test showed a significant correlation between EMT levels and MRP2 (+) CTCs grade *(R* = 0.341, *P* = 0.013) and high MRP2 expression *(R* = 0.283, *P* = 0.042) (**Table 8**), but no significant correlation between EMT levels and MRP2 low expression (*R* = -0.01, *P* = 0.945). In addition, these correlation scatter plots show how different CTC subtypes (Epithelial, Hybrid, Mesenchymal, (H+M)) relate to their MRP2 expression. All have strong positive correlations: Epithelial (*R* = 0.98, *P* < 2.2e−16; **Figure 3A**), Hybrid (*R* = 0.97, *P* < 2.2e−16; **Figure 3B**), Mesenchymal (R = 0.89, *P* < 2.2e−16; **Figure 3C**), and (H+M) (*R* = 0.97, *P* < 2.2e−16; **Figure 3D**). Therefore, MRP2 expression is closely related to CTC subtype counts, providing a new perspective for further investigation of the mechanisms of drug resistance in breast cancer.

**Table 8:** The correlation analysis between EMT grade and MRP2 expression grade in CTCs.

Grade	The number of patients in each EMT grade(X)	The number of patients in each MRP2 (+) CTCs grade (Y1)	The number of patients with different low MRP2 expression grade (Y2)	The number of patients with different high MRP2 expression grade (Y3)	X and Y1	X and Y2	X and Y3
G1	8	3	19	12	R = 0.341 *P* = 0.013	R = -0.010 *P* = 0.945	R = 0.283 *P* = 0.042
G2	13	2	21	11			
G3	12	8	12	12			
G4	19	39	0	17			

## 4 Discussion

Breast cancer metastasis remains a major obstacle to improving patient prognosis, with circulating tumor cells and multidrug resistance being critical drivers of treatment failure (1, 6, 7). This single-institution study pioneers the exploration of the MRP2-CTCs-EMT axis in breast cancer, aiming to clarify the role of MRP2 in CTC biology and its association with the EMT. Our key findings demonstrate that MRP2 expression in CTCs is closely linked to CTC subtype distribution, tumor progression, and EMT status, providing novel insights into the mechanisms underlying metastatic dissemination and drug resistance.

Mistry et al. (14) investigated how ABCC2 (MRP2) genotype and low BMI affect breast cancer patients’ clinical responses to sequential anthracycline-taxane chemotherapy. Their key finding was that specific ABCC2 genetic variants are linked to lower chemotherapy efficacy, which directly matches the focus of our study on ABCC2-mediated multidrug resistance (MDR) in CTCs. Alanazi et al. (15) analyzed ATP-binding cassette transporter genomic alterations (e.g., mutations, amplifications) and expression patterns, reporting that ABCC2 dysregulation correlates with poor survival in breast and prostate cancer. This study reinforces ABCC2’s prognostic relevance, which we link to our CTC-specific findings. The high CTC detection rate (94.2%) in our cohort aligns with previous observations that CTCs are prevalent in patients with breast cancer, underscoring their potential as liquid biopsy markers (16, 17). Notably, we found that H-type CTCs and MRP2 (+) CTCs were significantly correlated with larger tumor size, suggesting that these cellular subsets may contribute to tumor progression. This association is biologically plausible because H-type CTCs, which exhibit hybrid epithelial/mesenchymal properties, are thought to possess both adhesive and migratory capabilities, enabling them to survive in circulation and initiate metastatic colonization (18, 19). Meanwhile, MRP2-mediated drug efflux can enhance CTC survival under selective pressure from microenvironmental stress or systemic therapy, facilitating tumor expansion (20, 21).

Maciejczyk et al. (22) is a foundational study demonstrating that ABCC2 localization to the nuclear envelope of breast carcinoma cells correlates with poor clinical outcomes (e.g., shorter progression-free survival). Also, our finding that ABCC2 is highly expressed in (H+M)-type CTCs aligns with Maciejczyk et al.’s conclusion that ABCC2 contributes to tumor aggressiveness. Moreover, this subtype-specific expression pattern suggests that EMT progression may upregulate MRP2, a mechanism supported by our observation of a positive correlation between EMT grade and MRP2 (+) CTCs grade. EMT is known to endow tumor cells with mesenchymal traits, such as increased motility and resistance to apoptosis. Our data extend this paradigm by linking EMT to enhanced MDR potential through MRP2 upregulation (23, 24). The strong positive correlations between all CTC subtypes and MRP2 expression further indicate that MRP2 may be a conserved adaptive feature across CTC populations, regardless of the epithelial or mesenchymal phenotype.

Our results build upon and extend the existing literature in several key ways. Previous studies have established MRP2 as an important mediator of MDR in solid tumors (9, 25), and have highlighted the role of EMT in CTC biology (26, 27). However, the interplay between MRP2, CTC subtype, and EMT in breast cancer remains unexplored. Although Lin et al. (28) reported MRP family expression in breast cancer cell lines, they did not investigate CTCs or EMT associations. Similarly, Stefanovic et al. (29) characterized CTC subtypes in metastatic breast cancer but did not assess the MRP2 expression. Our study fills this gap by demonstrating a functional axis in which EMT status modulates MRP2 expression in CTCs, potentially enhancing their survival and metastatic capacity. This novel association provides a mechanistic explanation for why certain CTC subsets are more resistant to therapy and more likely to drive metastasis than others.

The clinical implications of our findings are substantial. MRP2 expression in CTCs, particularly in the H-type- and M-type subsets, could serve as a predictive biomarker for tumor progression and drug resistance. Patients with high MRP2 (+) CTC counts and advanced EMT grades may benefit from combined strategies targeting both EMT and MRP2-mediated efflux. For example, EMT inhibitors (e.g., TGF-β antagonists) can be used in conjunction with MRP2 blockers (e.g., probenecid) to sensitize CTCs to chemotherapy (21). Additionally, the correlation between MRP2 (+) CTCs and larger tumor size suggests that MRP2 may be a therapeutic target to prevent tumor growth and dissemination. Longitudinal monitoring of MRP2 expression in CTCs may also enable personalized treatment adjustments and improve clinical decision-making.

Despite these insights, our study has some limitations that warrant consideration. First, the sample size was relatively small (52 patients), and the single-institution design may limit the generalizability of our findings. Multicenter studies with larger cohorts are needed to validate the MRP2-CTCs-EMT axis in diverse patient populations. Second, we did not assess the prognostic significance of MRP2 (+) CTCs or their association with treatment response, which is critical for translating these findings into clinical practice. Future studies should correlate MRP2 expression in CTCs with clinical outcomes, such as progression-free survival and overall survival. Third, our analysis focused on mRNA expression, and functional studies (e.g., *in vitro* CTC culture and MRP2 knockdown experiments) are required to mechanistically validate the role of MRP2 in CTC survival and EMT regulation. Finally, the lack of data on pre- and post-treatment CTC dynamics prevented us from evaluating the effect of therapy on MRP2 expression in CTCs.

In conclusion, this study identifies a novel MRP2-CTCs-EMT axis in breast cancer, highlighting MRP2 as a key player in CTC biology and in EMT-associated drug resistance. Our findings provide a theoretical basis for developing combined targeting strategies that simultaneously disrupt EMT and MRP2-mediated efflux, offering new hope for improving the management of metastatic breast cancer. Future research should focus on validating these associations in larger cohorts, exploring the molecular mechanisms linking EMT to MRP2 upregulation, and evaluating the efficacy of MRP2-EMT targeted therapies in preclinical and clinical settings.

## Data Availability

The original contributions presented in the study are included in the article/**Supplementary Material**. Further inquiries can be directed to the corresponding author.
