# Supplementary material for: Expression patterns of MRP2 in circulating tumor cells of breast cancer: a single-institution study
Source: Front Oncol. 2025 Sep 23;15:1648842. doi: 10.3389/fonc.2025.1648842 (PMC12500547; doi:10.3389/fonc.2025.1648842)
Supplement: Supplementary file 1 [file Presentation1.pdf]

深圳市人民医院科研伦理委员会

审查意见函

声明：本伦理审查委员会遵从有关法规组成和工作，其审查和工作过程不受伦理审查委员会以外任何组织及个人的影响。

意见号：LL-KY-2024078-01

<table>
<tr><td>项目名称</td><td colspan="3">一项关于乳腺癌循环肿瘤细胞中 MRP2 的表达模式的单中心研究</td></tr>
<tr><td>主要研究者</td><td>周文斌</td><td>所在科室</td><td>甲乳外科</td></tr>
<tr><td>审查和批准文件</td><td colspan="3">1.临床研究立项申请学术审查意见表
2.科研项目开展前初始审查申请表
3.项目负责人履历
4.项目负责人资质证明材料
5.项目负责人责任声明
6.研究团队职责分工表
7.项目风险预评估及处置
8.试验方案　（版本号：2.0，版本日期：2024 年 01 月 20 日）
9.知情同意书　（版本号：2.0，版本日期：2024 年 01 月 20 日）</td></tr>
<tr><td>审查类别</td><td colspan="3">初始审查</td></tr>
<tr><td>审查方式</td><td colspan="3">☐会议审查　■简易审查　☐紧急会议审查</td></tr>
<tr><td>审查意见</td><td colspan="3">审查决定：必要修改后同意（简审）
根据卫生部《涉及人的生物医学研究伦理审查办法》（2016 年）、WMA《赫尔辛基宣言》和 CIOMS《人体生物医学研究国际道德指南》的伦理原则。经本伦理委员会审查，意见如下：

（一）1、知情同意书第五条中的内容请斟酌后明确，参加研究一定会受益吗？
2、知情同意书第九条第一点参加本研究不需要承担任何费用还是不需要承担基因检测费用。
3、知情同意书要明确样本的存放地点及期限，以及处理方式。
（二）审查结果为：必要修改后同意（简审）</td></tr>
<tr><td>主任委员
（被授权者）签名</td><td colspan="3">梁真
深圳市人民医院临床研究伦理委员会（盖章）
2024 年 05 月 13 日
深圳市人民医院 临床研究伦理委员会</td></tr>
<tr><td>备注</td><td colspan="3">1、请遵循临床试验伦理原则、遵循伦理委员会批准的方案开展临床研究，保护受试者的健康和权利。
2、对研究方案、知情同意书、招募材料等的任何修改，请提交修正案审查申请。
3、发生 SAE，请及时提交严重不良事件报告。
4、如有不依从/违背方案的情况，请及时提交违背方案报告。
5、请根据年度/定期跟踪审查频率，及时提交研究进展报告。</td></tr>
</table>

	6、暂停或提前终止临床研究，请及时提交暂停/终止研究报告。<br>7、完成临床研究，请提交结题报告。

地址：深圳市东门北路 1017 号 2 栋 2 楼 212 伦理办公室；

邮编：518020；　电话：0755-22943881；联系人：骆老师；邮箱：195323995@qq.com
