# Supplementary material for: Expression patterns of MRP2 in circulating tumor cells of breast cancer: a single-institution study
Source: Front Oncol. 2025 Sep 23;15:1648842. doi: 10.3389/fonc.2025.1648842 (PMC12500547; doi:10.3389/fonc.2025.1648842)
Supplement: Supplementary file 2 [file Presentation2.pdf]

受试者知情同意书

项目名称：乳腺癌转移相关循环肿瘤细胞中多药耐药相关蛋白2（MRP2）的研究

尊敬的受试者：

我们邀请您参加一项研究，该本研究已经得到深圳市人民医院临床研究伦理 委员会的审查和同意。在您作决定之前，我们希望您能了解开展本研究的原因以 及它需要您做的事情。您参加本研究纯属自愿，也就是说您既可以选择参加，也 可以选择不参加。研究团队将会为您讲解本信息告知书，并解答您的任何疑问。

如果您有任何不清楚的地方，请向我们提问。欢迎您与伴侣、家人、朋友和 医生等与您关系亲近的人讨论本研究及本文件所含的信息。在您考虑了与本研究相关的所有信息，并且您的所有问题都得到解答后，如 果您同意参加，则在进行任何研究相关的程序之前，研究团队会请您签署知情同 意书。

一、研究背景

乳腺癌是一种常见的恶性肿瘤，其转移是导致患者预后不良的主要原因之一。近年来，研究表明，CTCs作为肿瘤转移的生物标志物，可能在乳腺癌的进展和治疗中发挥重要作用。MRP2作为一种多药耐药相关蛋白，可能与乳腺癌细胞对治疗的耐药性相关。因此，了解MRP2在CTCs中的表达水平及其与乳腺癌转移的关系将有助于我们更好地理解乳腺癌的生物学特性，并为临床提供新的治疗靶点。

二、研究目的

本研究的研究目的是：我们旨在探讨循环肿瘤细胞（CTCs）中多药耐药相关蛋白2（MRP2）的表达与乳腺癌转移之间的关系，以期为乳腺癌患者的治疗提供新的生物学特征和临床意义。

三、研究过程

1.多少人将参与这项研究？

大约(　　60　　)人会在本院参与本研究。

2．研究持续时间

研究将持续大约 20个月。您可以在任何时间选择退出研究而不受到任何惩罚，也不会丧失您本应获得的任何利益。然而，如果在研究途中您决定退出本研究，我们鼓励您先和您的医生商议。

3．研究步骤

从2022年9月到2024年5月，在深圳市人民医院（中国）确诊的57例乳腺癌病例被选中进行CTCs检测和MRP2蛋白定量。收集了患者的临床特征，包括年龄、性别、肿瘤大小、ER、PR、HER-2及其他临床病理特征。

为避免皮肤静脉穿刺引起的细胞污染，首先丢弃2 mL外周血，然后将5 mL血液收集到乙二胺四乙酸（EDTA）管中（贝克顿 •迪金森，上海，中国）。在采血后4小时内使用Canpatrol系统（SurExam Biotech，广州，中国）进行检测。

四、风险和/或不适

本研究属于观察性研究,可能存在信息安全方面的风险。我们会尽全力保护您提供的信息不被泄露，然而，我们并不能保证信息的绝对安全。本研究中我们 所问您的一些问题可能会让您感到不舒服，你可以拒绝回答此类问题，同时， 研 究过程中您随时都可以休息。在研究中任何时刻，您都可以退出本研究。

研究相关程序风险如下:（若有相关检查可如下列举风险：）

•血液采集：血液样本将通过插入您的手臂或手部静脉内的小针头采集。在 插入和拔出针头时您可能会感到不适和/或酸痛，并有可能出现小的瘀伤。在极少数情况下，静脉可能堵塞，或者可能发生小的神经损伤，导致麻木和疼痛。如 果发生这种情况，一段时间后会消退。

五、参加研究有什么收益？

参加本研究能给您带来医疗获益。但我们希望通过您的参加将有助于为和您 一样患有同样病情/疾病的病人扩展对乳腺癌的认识，为将来该疾病的诊疗提供 更多的信息。

六、备选的治疗方案

本研究将可能可以提供其他治疗措施，您的诊断和治疗将由研究医生根据常 规实践自行判断，您可以继续维持本来的常规治疗方案。

七、研究结果的使用和个人信息的保密

当研究结束时，我们将对数据进行分析。您将有机会获知研究结果。您可以 向您的研究医生询问研究结果，并请他们作出解释。本研究的结果也可能在期刊 上发表，可能在会议上报告，但不会包含任何可能识别您身份的信息。为保证隐私，出于研究目的发布的记录不会附上您的姓名及其它身份识别信息。相反，您的信息将仅通过一个代码标识。只有研究医生和授权人员能够通过 一份清单把此代码与您的姓名联系起来，该清单将在研究中心安全地保存。可能为了确保研究是否在研究中心正确进行，必要时申办者、伦理审查委员 会以及政府管理部门按规定可以查阅您的资料，他们受保密义务约束，不会侵犯您的隐私。您有权控制对您个人信息的使用和披露。在国家法律允许的情况下，您可以 随时要求查看您的医疗信息。您有权通过研究医生查看所收集的有关您的所有信 息，并要求进行纠正。

八、研究相关新信息

研究期间，若有研究程序变化、新发现的副作用或可能影响您健康或参加意 愿的重大情况出现，研究小组将通知您。研究医生会立即通知您，还会与您一起讨论您是否想继续参加本研究。

九、关于研究费用、补偿以及损害赔偿

1. 研究相关检查费用及补偿

参加本研究您需要承担CTC检测费用。本研究也不会给予您任何补偿。

2. 损害赔偿

本研究将不提供任何治疗措施，您的诊断和治疗将由研究医生根据常规实践 自行判断，若您确因参加研究而受到损害，您可以获得由 深圳市人民医院 提供的免费治疗，并将依法进行赔偿。

十、受试者的权利和责任

1. 您的权利

在参加研究的整个过程中，您都是自愿的。如果您决定不参加本研究，也不 会影响您应该得到的其他治疗。如果您决定参加，会要求您在这份书面知情同意 书上签字。您有权在试验的任何阶段随时退出试验而不会遭到歧视或受到不公平 的待遇，您相应医疗待遇与权益不受影响。

2. 您的责任

参与研究，请您遵守以下约定：

- 如您想终止临床研究，任何时候您都可以告诉您的研究医生
- 需要提供有关自身病史和当前身体状况的真实情况
- 遵从研究人员的指示
- 告诉研究医生自己在本次研究期间所发现的任何不适。

十一、相关联系方式

如果您有与本研究相关的任何问题，请通过电话 15218227975 与 关家裕 联系。

受试者签字页

知情同意声明

我已被告知此项研究的目的、背景、过程、风险及获益等情况。我有足[illegible]的时间和机会进行提问，问题的答复我很满意。

我也被告知，当我有问题、想反映困难、顾虑、对研究的建议，或想进[illegible]步获得信息，或为研究提供帮助时，应当与谁联系。

我知道我可以选择不参加此项研究，或在研究期间的任何时候无需任何理[illegible]退出本研究。此外，研究者没有对我使用欺骗、利诱、胁迫等手段强行让我[illegible]意参加研究。

我已知道如果我的状况更差了，或者我出现严重的不良反应，或者我的[illegible]究医生觉得继续参加研究不符合我的最佳利益，他/她会决定让我退出研[illegible]。无需征得我的同意，资助方或者监管机构也可能在研究期间终止研究。如[illegible]发生该情况，医生将及时通知我，研究医生也会与我讨论我的其他选择。

我已经阅读这份知情同意书，并且同意参加本研究。

受试者签名：[illegible] 雷玉芳 [illegible] [illegible] 吴明国 [illegible] [illegible] 邓永来 王如敏 [illegible] 李智华 [illegible] [illegible] [illegible] 廖玉霞 郑若兰 马文兰 [illegible] 欧阳翠霞 李恒 [illegible] [illegible] [illegible] [illegible] [illegible] [illegible] [illegible] [illegible] [illegible] [illegible] 金莉 刘玉莲 林丽君 [illegible] [illegible] 张果 [illegible] 司海芸 [illegible] 李智华 徐萌 [illegible] [illegible] [illegible] [illegible] [illegible] [illegible] 刘惠 [illegible]
